# Supplementary material for: The WT1-like transcription factor Klumpfuss maintains lineage commitment of enterocyte progenitors in the Drosophila intestine
Source: Nat Commun. 2019 Sep 11;10:4123. doi: 10.1038/s41467-019-12003-0 (PMC6739418; doi:10.1038/s41467-019-12003-0)
Supplement: Supplementary file 1 — Supplementary Information [file 41467_2019_12003_MOESM1_ESM.pdf]

Supplementary Information for

The WT1-like transcription factor *Klumpfuss* maintains lineage commitment of enterocyte progenitors in the *Drosophila* intestine

Jerome Korzelius[1*§], Sina Azami[1*], Tal Ronnen-Oron[2], Philipp Koch[1], Maik Baldauf[1], Elke Meier[1], Imilce A. Rodriguez-Fernandez[3], Marco Groth[1], Pedro Sousa-Victor[2], and Heinrich Jasper[1,2,3§]

1. Leibniz Institute on Aging – Fritz Lipmann Institute (FLI), Jena, Germany
2. Buck Institute for Research on Aging, 8001 Redwood Boulevard, Novato, CA 94945-1400, USA
3. Immunology Discovery, Genentech, Inc., 1 DNA Way, South San Francisco, CA 94080, USA

*Present address: Max-Planck-Institute for Biology of Aging, Cologne, Germany

§ (Co-)Corresponding author

jkorzelius@age.mpg.de jasper.heinrich@gene.com

Supplementary Figures

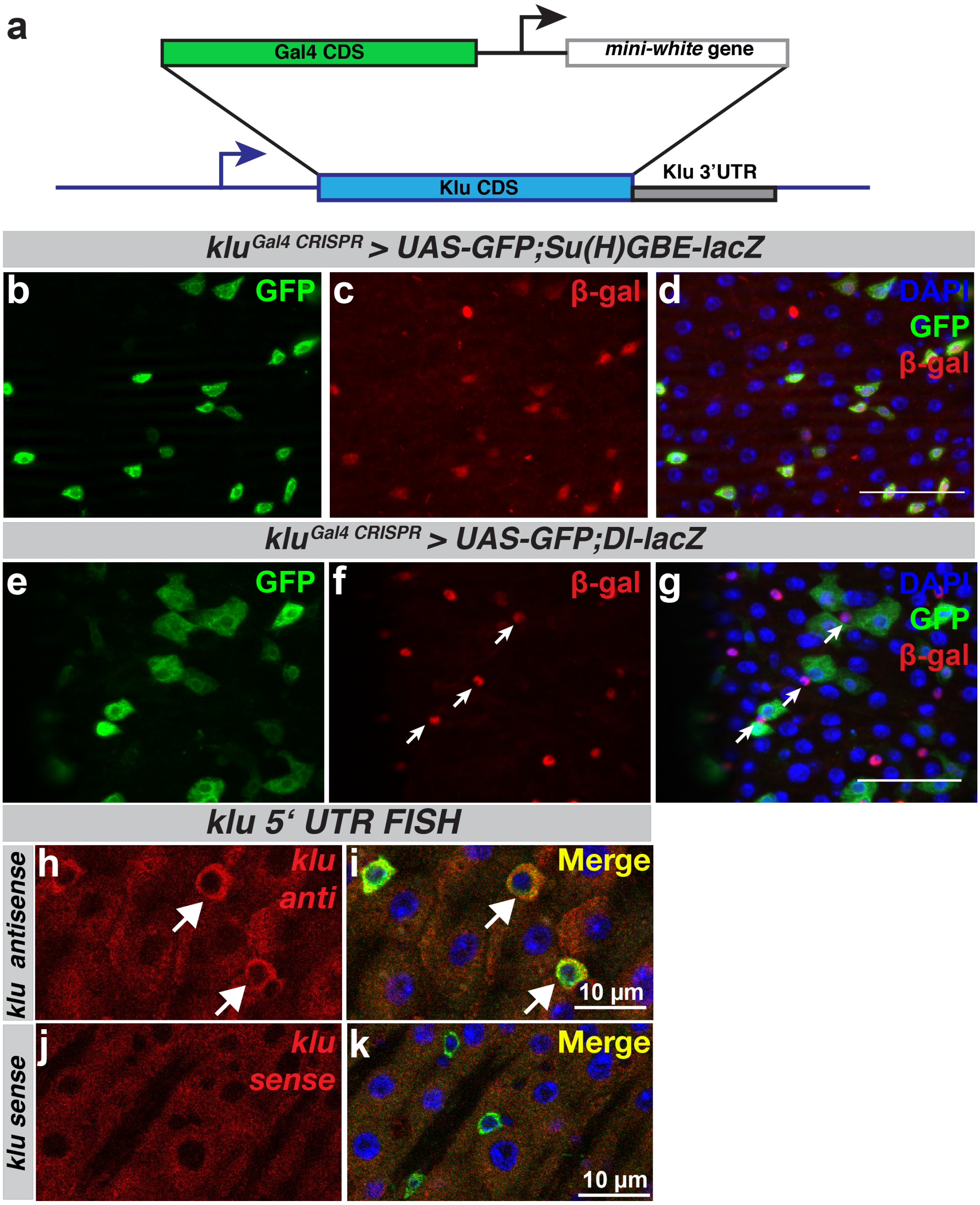

Supplementary Figure 1. A *klu-Gal4* CRISPR knock-in allele recapitulates the EB-specific expression pattern of Klu. a. Diagram of the CRISPR strategy for replacing the klu CDs with a Gal4 CDS and mini-white reporter gene. **b-g**. Animals carrying a *klu*$^{Gal4\ CRISPR}$ knock-in construct were combined with UAS-GFP and either the EB-reporter *Su(H)GBE-lacZ* (**b-d**) or the ISC-marker *Dl-lacZ* (**e-g**). Note that *klu-Gal4> UAS-GFP* expression overlaps with the EB-marker *Su(H)GBE-lacZ*, but not with the Dl-lacZ-positive ISCs, which are found adjacent to the *klu-Gal4> UAS-GFP*-expressing cells (**f,g**, arrows).

Representative areas of posterior midgut are shown. $n = 4$ animals for (**b-d**) $n = 3$ animals for (**e-g**). **h,i**. A *klu* antisense FISH probe showed strong staining of EBs (arrows, green: marked with *Su(H)GBE-Gal4 > UAS-GFP*, GFP antibody staining). **j,k**. A *klu* sense control probe did not show specific staining. Representative areas of posterior midgut are shown. $n = 3$ animals/genotype. Scale bar = 50 μm, except in **i,k**: scale bar = 10 μm.

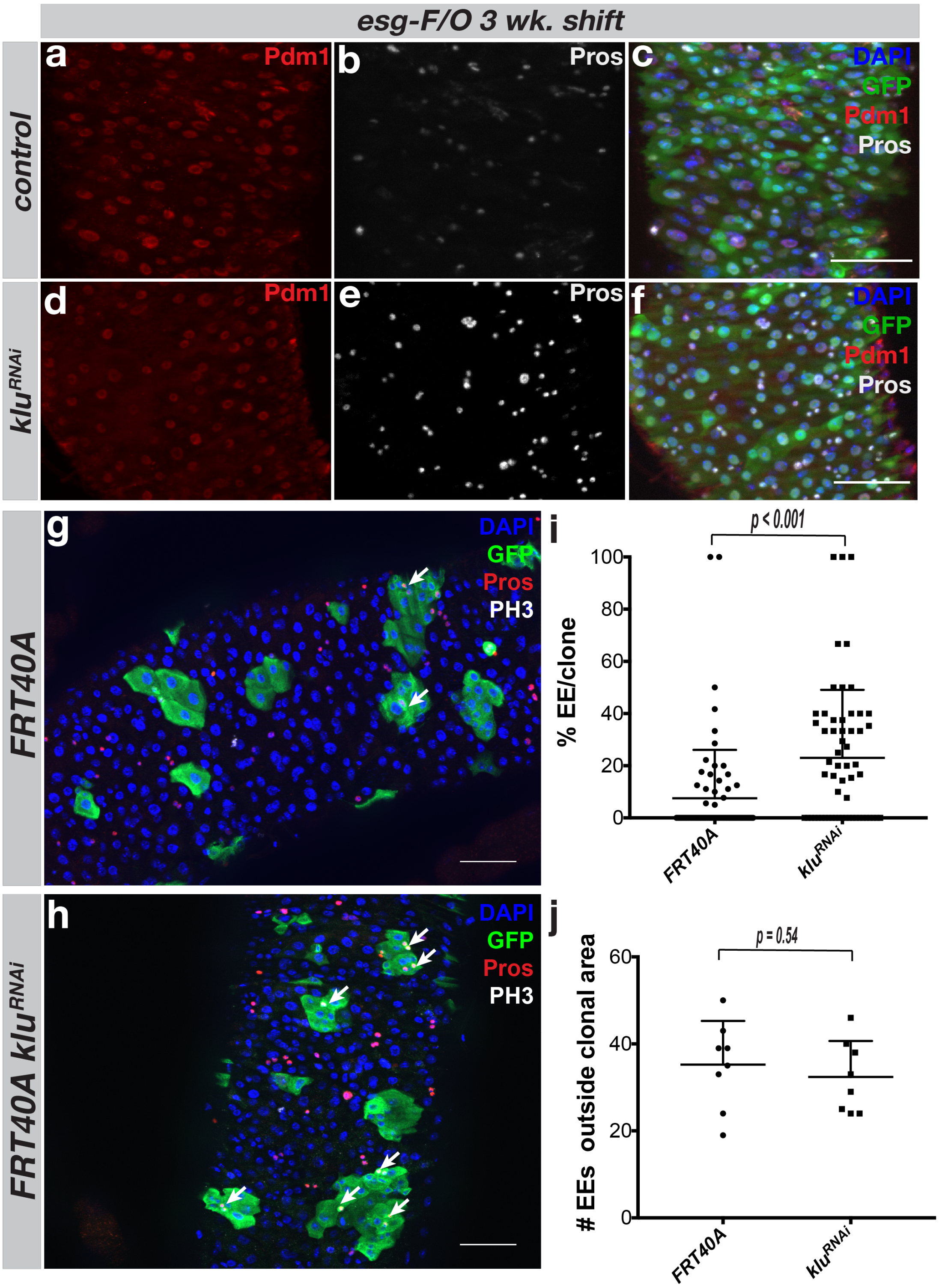

Supplementary Figure 2. Loss of Klu results in a shift in the EC-to EE ratio, but not to misdifferentiation of ECs. a-c. Control *esg-F/O* clones consist of Pdm1-positive enterocytes (**a**) and Pros-positive EE cells (**b**). **d-f**. Expression of *klu^{RNAi}* increased the ratio of Pros-positive cells (**e**), but Pdm1-positive ECs can still form in these clones (**d**). Representative areas of posterior midgut are shown. *n* = 3 animals/genotype. **g-j**. MARCM40A clones 7 days after clonal induction **g**. Control *FRT40A* clones have on average 7.5 % EEs/clone. **h**. *klu^{RNAi}* MARCM clones contain on average 23% EE's/clone 7 days after clonal induction (arrows, quantification in **i**). Notably, the amount of EE cells in surrounding non-clonal tissue does not significantly change (**j**). Representative areas of posterior midgut are shown. Error bars represent mean +/- S.D. Significance was calculated using Student's t-test with Welch's correction. Scale bar = 50 μm

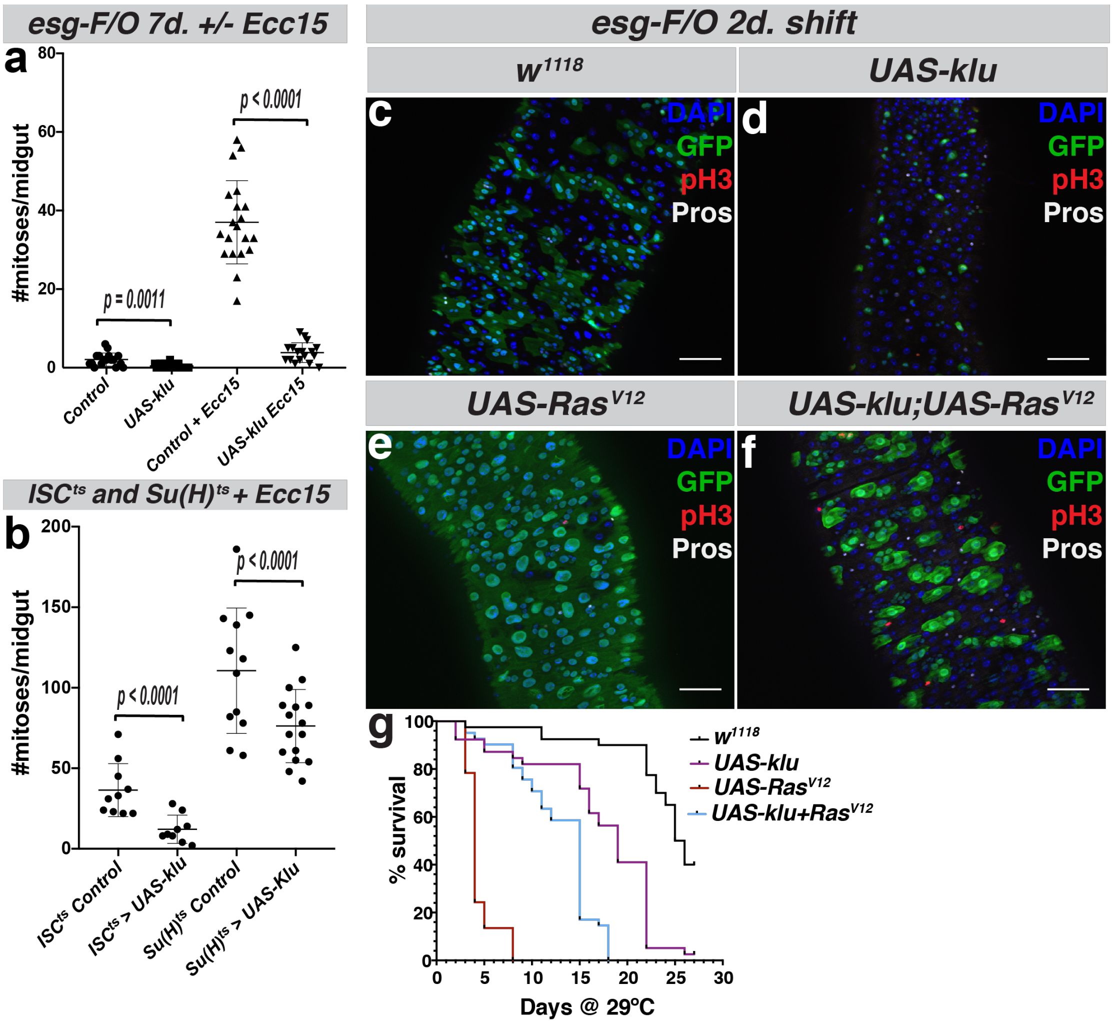

Supplementary Figure 3. Klu overactivation represses Ras^{V12}-induced overgrowth. a. Quantification of mitosis in midguts of *esg-F/O* animals expressing *UAS-klu* in the presence or absence of *Ecc15* infection. *n* = 17 for control uninfected, *n* = 21 for *UAS-klu* uninfected, *n* = 20 for control+*Ecc15*, *n* = 17 for *UAS-klu+Ecc15*. **b**. Quantification of mitosis in midguts of control and

UAS-klu ISC^{ts} and *Su(H)^{ts}* lines upon *Ecc15* infection. n = 10 for *ISC^{ts}* control, n = 9 for *ISC^{ts} Ecc15*, n = 12 for *Su(H)^{ts}* control and n = 16 for *Su(H)^{ts} Ecc15*. For **a,b**: Error bars represent mean +/- S.D. Significance was calculated using Student's t-test with Welch's correction. **c-g**. Klu overactivation represses RasV12-induced overgrowth. **c**. After 2 days of induction, control *esg-F/O* clones were mostly 1-2 cell clones, similar to *UAS-klu*-expressing clones (**d**). **e.** *esg-F/O* > *UAS-Ras^{V12}* occupied the complete intestine 2 days after induction. **f**. Co-expression of *UAS-klu* with *UAS-Ras^{V12}* markedly reduced Ras^{V12}-induced overgrowth. Representative areas of posterior midgut are shown. n = 4 animals for (**d**), n = 3 animals for (**c,e,f**). **g**. Survival assay of *esg-F/O* flies kept at 29°C expressing the abovementioned constructs in an independent experiment. n= 40 control, n= 39 *UAS-klu*, n= 37 *UAS-Ras^{V12}* and n=41 *UAS-Ras^{V12};UAS-klu*. Scale bar = 50 μm.

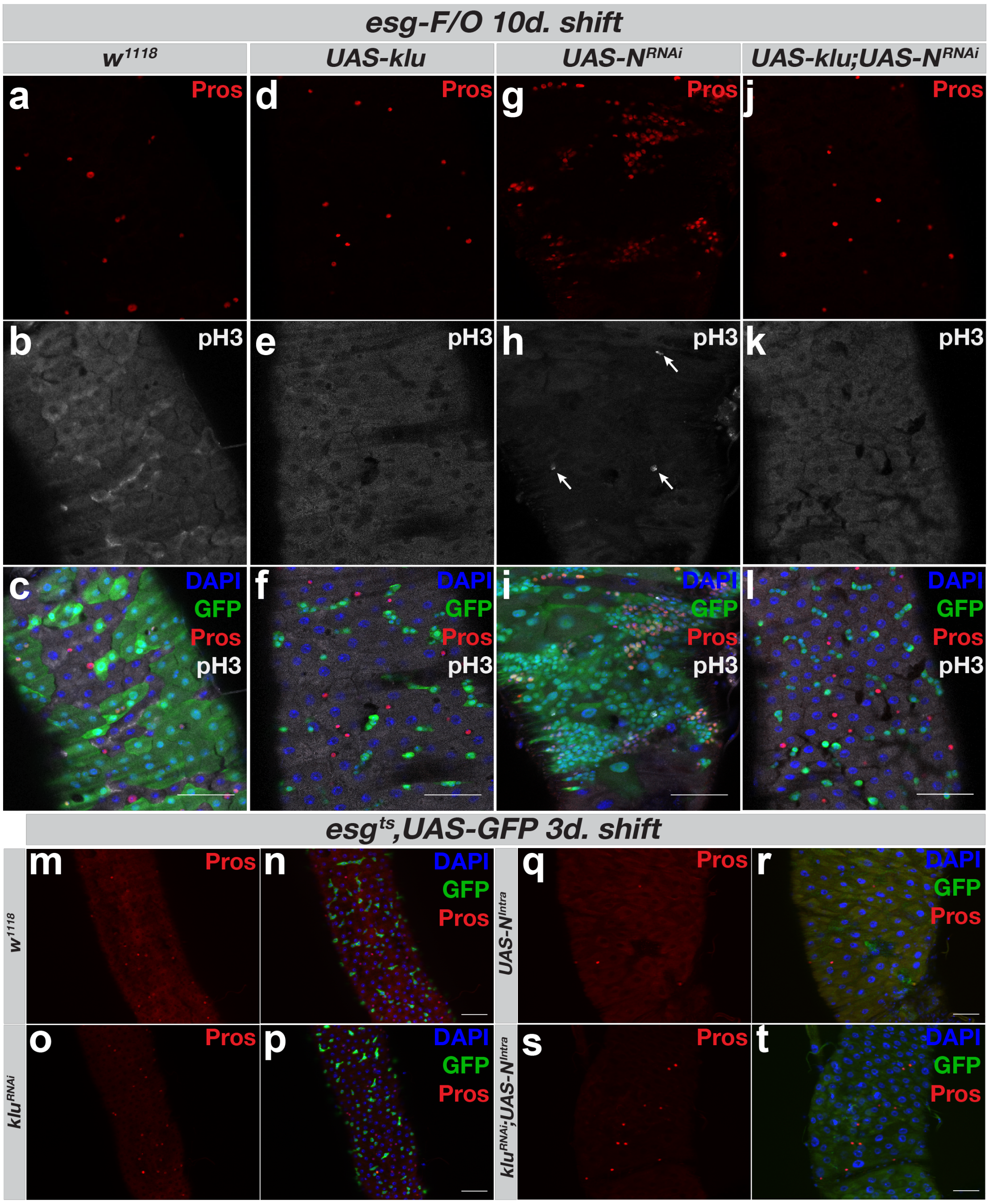

Supplementary Figure 4. Klu acts downstream of Notch in N^{RNAi}-induced tumor formation in the intestine. a-c. Control *esg-F/O* clones differentiated into ECs and EEs and occupied most of the posterior midgut 10 days after clonal induction **d-f**. *UAS-klu* clones fail to proliferate or to differentiate. **g-i**. *esg-F/O>N^{RNAi}* clones form tumors consisting of mitotic ISC-like cells (**h**, arrows) and Pros-positive EE cells (**g**). **j-l**. *esg-F/O>N^{RNAi};UAS-klu* clones did not overproliferate and resembled *UAS-klu* clones. Representative areas of posterior midgut are shown. $n = 3$ animals for (**a-l**). **m,n**. Klu knockdown cannot repress the ability of constitutively active Notch to induce EC-differentiation. **m,n**. Control *esg^{ts},UAS-GFP* cells 3 days after induction at

29°C. **o,p**. *klu*^{RNAi} expression in *esg*^{ts}*, UAS-GFP* cells. **q-r.** Expression of constitutively active Notch (*UAS-N*^{intra}) leads to loss of the Esg^{+} compartment due to premature differentiation into ECs. **s,t**. *klu*^{RNAi} combined with *UAS-N*^{intra} is not sufficient to repress the premature EC-differentiation phenotype seen in *UAS-N*^{intra}. Representative areas of posterior midgut are shown. *n* = 3 animals for (**m-t**) Scale bar = 50 μm.

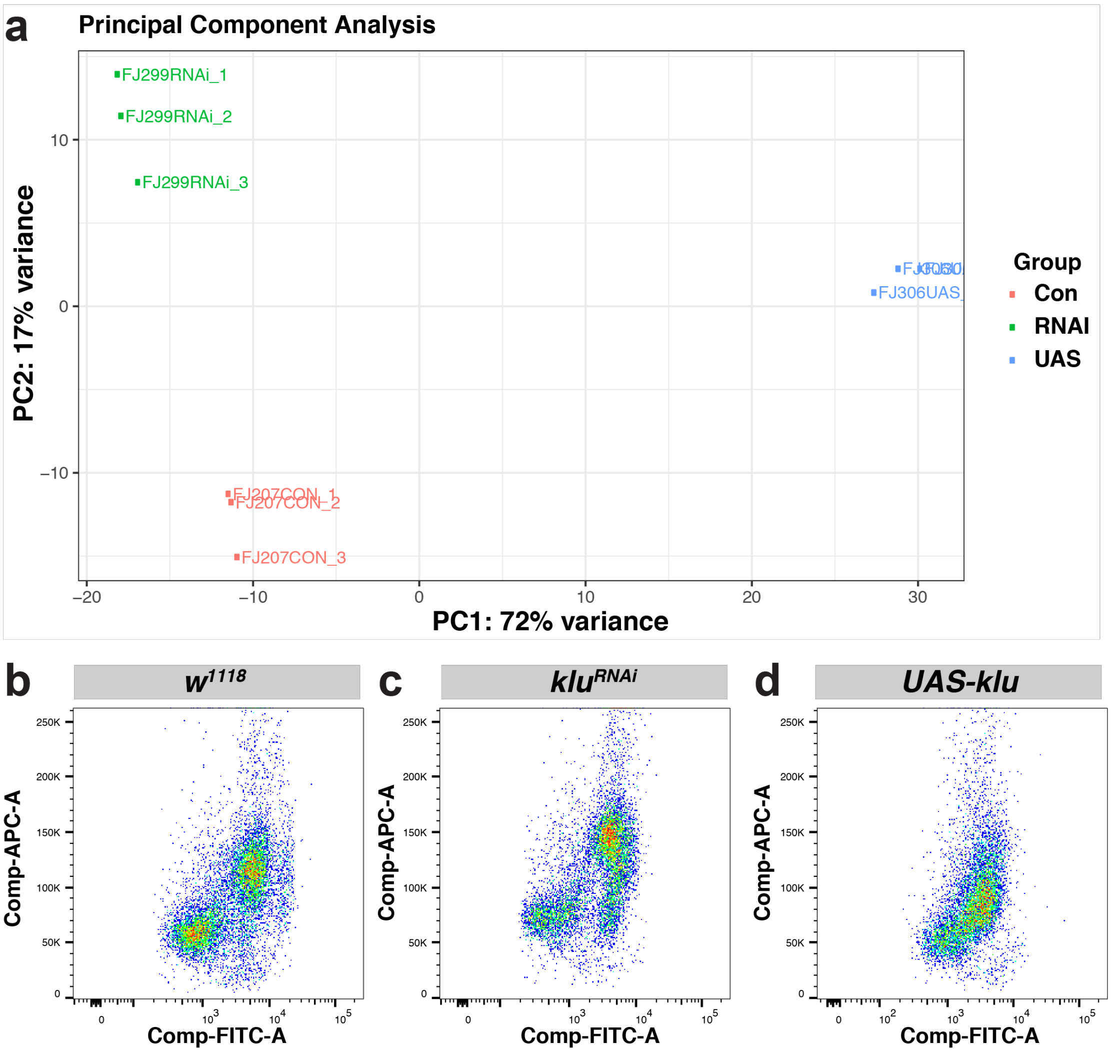

Supplementary Figure 5. PCA-analysis plot of RNA-Seq samples of control *esg*^{ts} animals or animals expressing either *UAS-klu* or *klu*^{RNAi}. a. Principal component analysis (PCA) revealed a close correlation between all biological replicates in PC1 and PC2. Note that UAS-klu samples cluster away from control (*esg*^{ts} X *w*^{1118}) and *klu*^{RNAi} samples in PC1 of the PCA-plot. **b-d.** FACS-sorting of *esg*^{ts}*, UAS-GFP* animals crossed to the indicated genotypes (see Methods). X-axis is GFP-intensity (FITC-A), Y-axis is DNA-intensity (APC-A: NuclearID Red DNA stain). Whereas there is little difference between the presumptive ISC (2N DNA, less GFP, bottom left) and EB (4N DNA, more GFP, top right) populations between control (**b**) and *klu*^{RNAi} samples (**c**), the *UAS-klu* sample shows a loss of these two clear sub-populations (**d**).

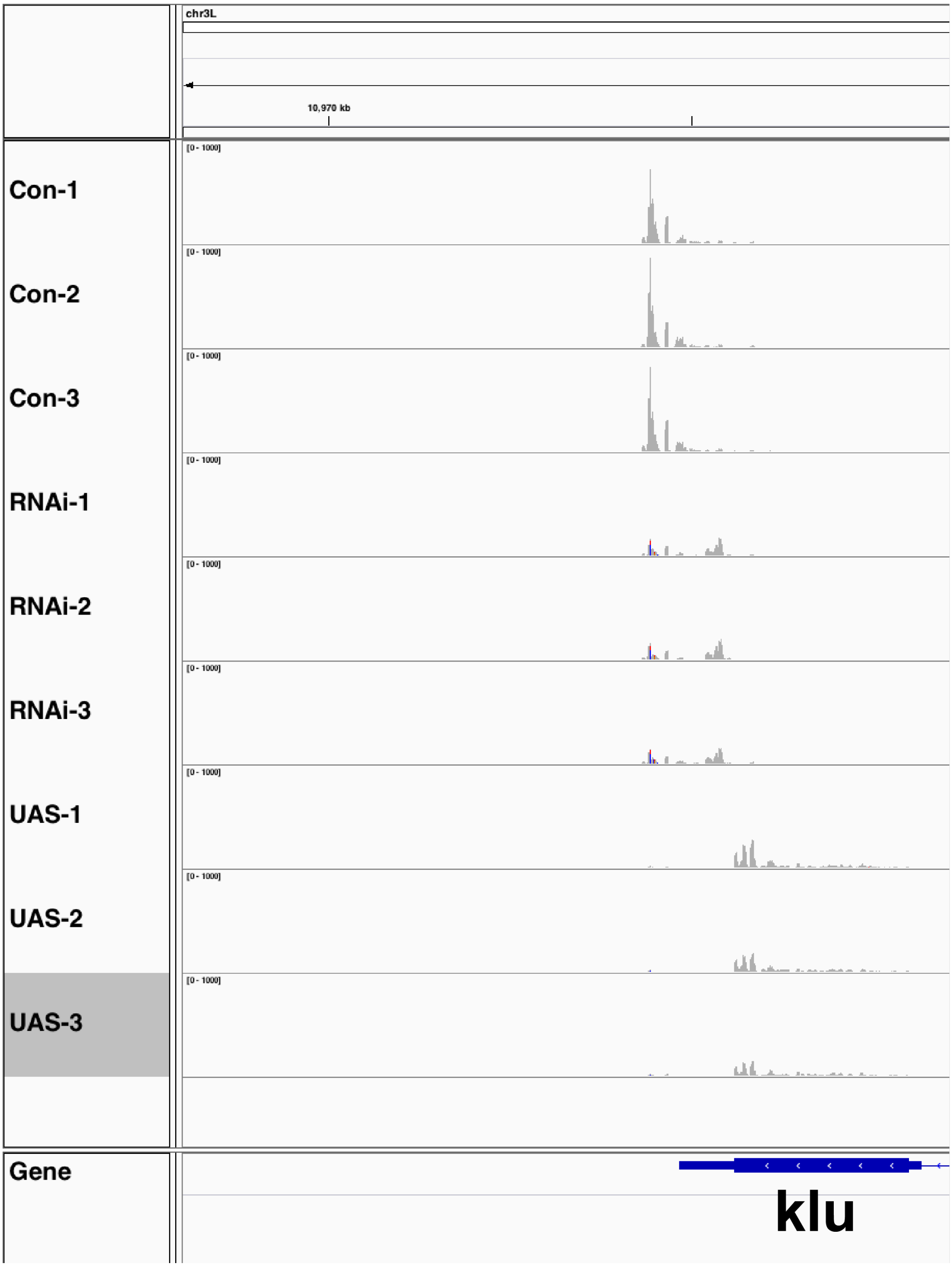

Supplementary Figure 6. IGV view of reads around the *klu* locus in all RNA-Seq samples. The number of reads near the 3' end of the *klu* gene is high in control Esg-positive sorted cells, but reduced in samples expressing klu^{RNAi}. *UAS-klu* samples show an absence of reads from the 3' end, but more reads along the coding region of the gene.

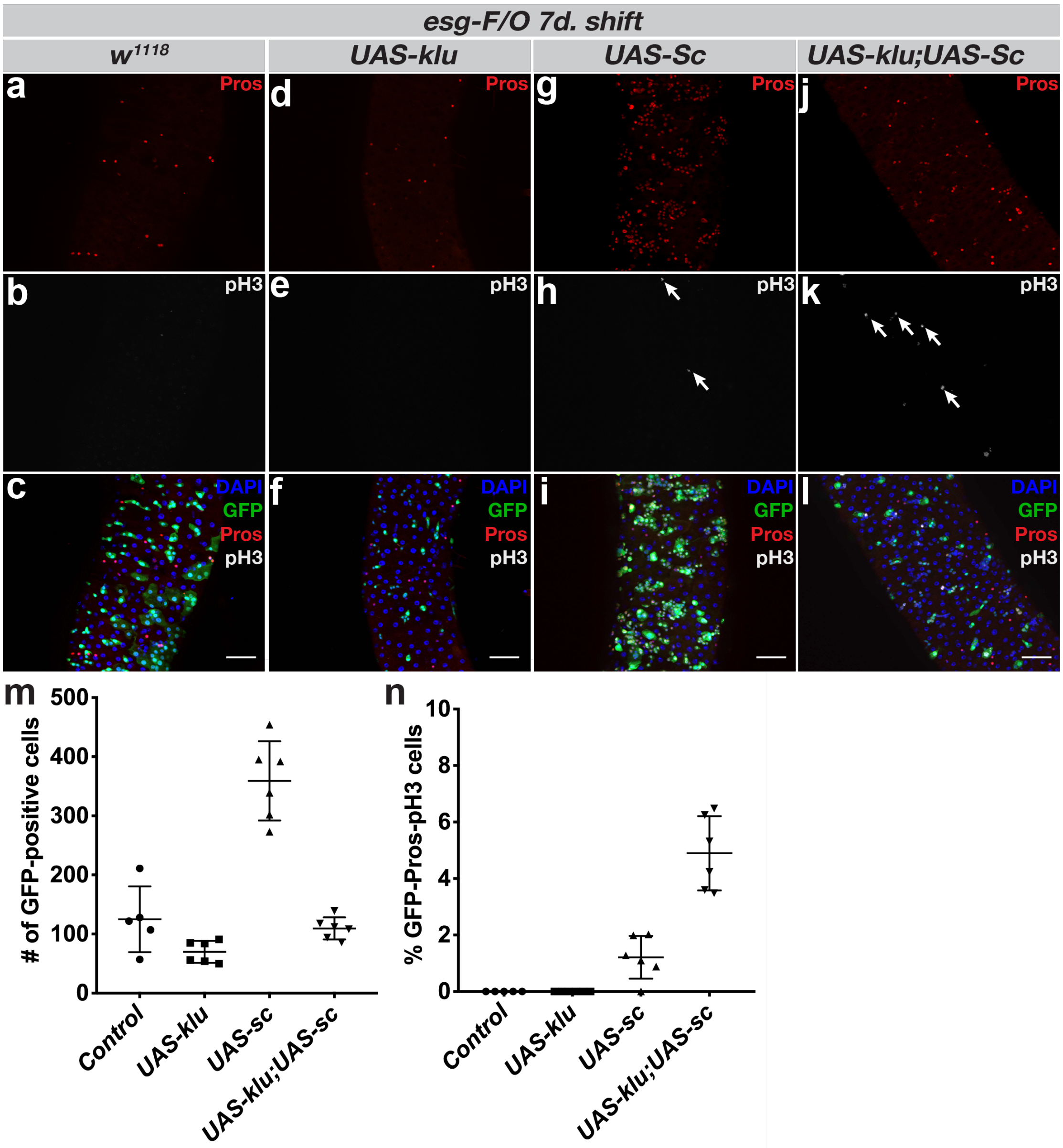

Supplementary Figure 7. Klu inhibits clonal proliferation, but not EE differentiation in *esg-F/O* clones. **a-c**. Control *esg-F/O* clones have approximately 5% EE cells, marked by Pros (red) 7 days after clonal induction. **d-f**. Ectopic *UAS-klu* expression completely blocks differentiation. **g-i.** *UAS-sc* overexpression results in clones consisting almost entirely of EE cells. *UAS-sc* clones also show increased numbers of mitotic cells (**h**, indicated by staining for pH3S10 in white, arrows). **j-l**. Co-expression of Klu and Scute results in clones that still have EE differentiation, albeit at a lesser rate as clones expressing only Scute (D). These clones contain fewer cells that *UAS-sc* clones, but more pH3S10-positive cells/clone (**k**, arrows). **m**. Quantification of $GFP^+/Pros^+/pH3^+$ triple-positive cells/clone of the genotypes in (**a-l**). **n**. Quantification of the total number of GFP^+ cells/ROI of the genotypes in (A-D). Error bars represent mean +/- S.D. Significance was

calculated using Student's t-test with Welch's correction. *n* = 5 for control, *n* = 6 for *UAS-klu, UAS-sc* and *UAS-klu;UAS-sc*. Scale bar = 50 μm.
