# Supplementary material for: The WT1-like transcription factor Klumpfuss maintains lineage commitment of enterocyte progenitors in the Drosophila intestine
Source: Nat Commun. 2019 Sep 11;10:4123. doi: 10.1038/s41467-019-12003-0 (PMC6739418; doi:10.1038/s41467-019-12003-0)
Supplement: Supplementary file 3 — Description of Additional Supplementary Files [file 41467_2019_12003_MOESM3_ESM.docx]

**Title:** **Supplemental Data File 1
RNA-Seq analysis of significantly regulated genes by Klu in Esg^+^ cells.**
DeSeq2 analysis of differentially expressed genes between control and *UAS-klu* as well as control and *klu^RNAi^* Esg^+^ cells (see Figure 5 and Methods for details). Column names: **FBgn ID**: Flybase gene name ID, **baseMean**: mean normalised counts, averaged over all samples from both conditions control (**CTRL**) VS treatment (**UAS** or **RNAi**), **Log2FoldChange**: base log2 fold-change in counts between control VS treatment **stat**: Wald statistic, **pvalue**: *p*-value for the statistical significance of this change, **Padj**: *p*-value adjusted for multiple testing through the Benjamini-Hochberg method, **symbol**: Flybase gene symbol. **UAS UP**: Upregulated in *UAS-klu* compared to Control, **UAS DOWN**: Downregulated in *UAS-klu* compared to Control, **RNAi DOWN**: Downregulated in *klu^RNAi^* compared to Control, **RNAi UP**: Upregulated in *klu^RNAi^* compared to Control.

**Title:** **Supplemental Data File 2
Python script for assignments of peaks from the damid-seq pipeline to genes.**

Your manuscript has been checked for clarity and against journal policies and formatting style. The issues listed below must be addressed. If using Microsoft Word, please use the tracked changes feature to make these changes.

***Please provide a point-by-point response to the list below with your submission.***


TITLE PAGE

* Please shorten the abstract to 150 words or fewer.

*We have abbreviated the abstract to 144 words in the final version* of the manuscript*.*

*Please change your title to specify you make these findings in Drosophila and for specification of the fate of enterocytes. Please note this should be a maximum of 15 words (with no punctuation).
We changed the title to **­­­“*The WT1-like transcription factor Klumpfuss maintains lineage commitment of enterocyte progenitors in the Drosophila intestine”*** (15 words) in the final version of the manuscript.

MAIN TEXT

* Please shorten the main manuscript text (Introduction, Results, and Discussion, not including figure legends or Methods) to approximately 5,000 words or fewer.

*We have abbreviated the Main Text to 5,000 words in the final version.*

* Please shorten all subheadings in the Results section to fewer than 60 characters including spaces.

*We have abbreviated the subheadings in the Main Text to fewer than 60 characters in the final version.*

*Please refer to Supplementary Fig x (not Figure Sx).

*We have applied these changes in the final version.*

*Please remove prime labels from Figure subpanel s (as this does not meet with the journal style) and instead, please use the next letter and cite correctly in the legend/your main text. This also applies to supplementary figures.
*We have applied these changes in the final version.*


LANGUAGE AND STYLE

* Please avoid using speech marks around words or phrases. In most cases they are unnecessary.

*We have applied these changes in the final version.*

* Wherever p-values are stated in the text and figure legends, please also state the name of the statistical test.

*We have applied these changes in the final version.*

*If you refer to significant changes in your results section text, please ensure this is supported by a p-value/statistical value or simply change the wording to “substantial”. *We have replaced “significant” with “substantial” where appropriate in the final version.*


METHODS AND DATA

*Please remove punctuation from Methods subheading titles.

*We have applied these changes in the final version.*

* In the Methods section, please provide sufficient information such that the experiments could reasonably be reproduced without reference to other papers, and avoid use of the term "as described previously".

*We have changed this in our final version, specifically for the “* ***Immunostaining and microscopy****” and ”****Midgut FACS, RNA-isolation and Sequencing****” subheadings of the Methods section.*

* For all studies developing new software or using custom code that is deemed central to the conclusions, a statement must be included, under the heading "Code Availability", indicating whether and how the code can be accessed, including any restrictions to access. This section should also include information on the versions of any software used, if relevant, and any specific variables or parameters used to generate, test, or process the current dataset. Code availability statements should be provided as a separate section after the Data Availability section but before the References. Please see our policy on code availability for more information. <http://www.nature.com/sdata/for-authors/editorial-and-publishing-policies>#code-avail

* Please separate your Data Availability and Code Availability statements into two separate sections, with the Code Availability section placed after the Data Availability section.
*We have applied these changes in the final version.*

*Please provide a separate Data availability statement. Please ensure that all accession code raw data are publicly available. GSE132243 does not appear to be – and please note: this needs to be publicly available before your file can be editorially accepted.

*We changed the headings in the final version and have released the RNA-Seq dataset GSE132243 for immediate access.*

*Please remove this statement: “Custom code for DamID analysis is available upon request to the corresponding author(s).” and provide all needed information/supplementary code (see above).

*We have removed this statement from the final version. We have added the custom script for peak annotation as Supplementary data file 2 in the submission.*

*Please place your reference list after your Methods section.

*Please specify what competing interests the two authors may have – as you currently state they are Genentech-Roche U.S.A. employees – does this relate to drugs used in the study or patents?

*As this work does not relate to any patents and/or drugs used in the study, we have changed this statement to "****The authors declare no competing interests****." in the final version.*


END NOTES

* Please supply an "Author Contributions" section after the Acknowledgement section that refers to all authors.

*We have added the name of Imilce A. Rodriguez-Fernandez (I.A.R-F.) to the Author Contribution section in the final version.*

* Please provide a "Competing Interests" section after the "Author Contributions" section that refers to all authors. If there are no competing interests, please add the statement "The authors declare no competing interests."

*As this work does not relate to any patents and/or drugs used in the study, we have changed this statement to "****The authors declare no competing interests****." in the final version.*

* Please ensure that all in-text citations to references (e.g. "Smith et al. show...") are followed by their corresponding reference citation number from the reference list, and that the references are numbered in the order they appear in the text (then tables and figures).

*We have applied these changes in the final version.*


DISPLAY ITEMS

* Please check whether your manuscript or Supplementary Information contain third-party images, such as figures from the literature, stock photos, clip art or commercial satellite and map data. We strongly discourage the use or adaptation of previously published images, but if this is unavoidable, please request the necessary rights documentation to re-use such material from the relevant copyright holders and return this to us when you submit your revised manuscript.
In particular, please indicate whether you or a co-author created all images in figure 5.

*All images in our revised Fig. 5 have been created by Jerome Korzelius in Adobe Illustrator.*

* Please ensure that figure legend titles are brief - they should not occupy more than one line in the final proof.

*We have substantially shortened all figure legend titles to fit the demands.*

*Please ensure that the fly (as shown in Figure 5A) is drawn by you and not taken from a stock image – as we are a commercial publisher and cannot publish some stock images.

*All images in our revised Fig. 5, in particular 5A., have been created by Jerome Korzelius in Adobe Illustrator.*

*For images, please ensure you comment on how representative the images are ie of how many images looked like this in x number of experiments

*We have added the statement “Representative areas of posterior midguts are shown” and numbers of animals analyzed to the figure legends where appropriate. As mentioned in the Figure Legends, the majority of immunofluorescence images represent samples, for which quantification is presented in a separate panel in the same figure.*

*In figure 7, you may wish to move panel f to a separate figure and discuss in more detail, as the model for your work.
*We have now moved the Fig.7F panel into a separate Fig. 8 as the model for our work*.

* Our format does not allow for subdivision of figure panels beyond 1a, b, c, etc. Please relabel the panels accordingly.

*We have modified our panels to a “a,b,c,”-format.*

* Please ensure that figure panels are labelled using an a, b, c... convention.

*We have modified our figures to a “a,b,c,”-format.*

* Please ensure that each display item is no larger than a single A4 portrait page (260x179 mm).

*All main Figures were created in Adobe Illustrator CC2019 in an A4-format (which is 210 x 297 mm, not 260x179mm).*

* Please ensure that at least one micrograph in each equivalent group in each figure is supplied with a representative scale bar, whose length is stated in the corresponding figure legend.

*We have checked all main and supplementary figures and provided the necessary scalebars for each figure/equivalent figure group.*

*Please ensure that the legend for figure 3 accurately describes the panels presented in the figure and ensure there are not two “C” labels. *We have adapted the figure legend for figure 3 to represent the current arrangement of panels and checked the labeling of individual panels.*

* Some figures in your paper include bar charts. Please overlay the corresponding data points (as dot plots) in the bar charts.

*We have changed the bar charts from* ***Fig.1, Fig.4 and Fig. 5*** *into either dot-plots or bar charts with points*

* Please define any new abbreviations, symbols or colours present in your figures in the associated legends. Please do not use symbols in your legend, instead please write out the symbols in words (blue circles, red dashed line, etc.). *We have changed this in our Figure Legends.*

* In each figure and supplementary figure where error bars are used, they must be defined. One statement at the end of each figure is sufficient if the error bars are equivalent throughout the figure.
*We have changed this in our Figure Legends.*

* Wherever statistics have been derived (e.g. error bars, box plots, statistical significance), the legend needs to provide and define the n number (i.e. the sample size used to derive statistics) as a precise value (not a range), using the wording "n=X biologically independent samples/animals/cells/independent experiments", etc. as applicable. *We have defined n-numbers for all experiments in our Figure Legends.*

**SUPPLEMENTARY INFORMATION**

* We do not edit Supplementary Information files; they will be uploaded with the published article as they are submitted with the final version of your manuscript. Any tracked changes should be removed from the file and the file should be provided as a PDF file. Supplementary Figures do not need to be provided separately.

*We have uploaded our Supplementary Information file containing 7 Supplementary Figures as a separate PDF-file.*

* The only section headings permitted in the Supplementary Information are Supplementary Figures, Supplementary Tables, Supplementary Methods, Supplementary Notes, Supplementary Discussion, Supplementary References. All other section headings and numbering should be removed or relabelled.

*We have adapted our Supplementary Information File accordingly.*

* In the Supplementary Information file and the main manuscript text, supplementary items must be labelled and cited using only the following formats: Supplementary Figure 1, Supplementary Table 1, Supplementary Methods, Supplementary Note 1, Supplementary Discussion, and Supplementary References. Please note the use of "Supplementary" and that we do not use the "S" prefix.

*We have applied these changes throughout our manuscript.*

* Please supply legends for each Supplementary Movie/Audio/Data file in your cover letter (not in the Supplementary Information file). Please label each files as Supplementary Movie/Audio/Data 1, etc.

*We have supplied two Supplementary Data Files, for which we included the Legends in our Cover letter (see above).*

* Large datasets should be supplied as Supplementary Data files, whereas smaller tables should be supplied as supplementary tables.

*See below*

*As the information in the current supplementary table 1 is rather long – and better viewed in an excel file, please upload as a supplementary dataset – preferably with each tab uploaded as a separate supplementary data file. In the top row/column of each file, please include the following information: Supplementary data X. <title>

*As Supplementary Table 1 is indeed quite long and has multiple tabs as the output of our DeSeq2 RNA-Seq analysis, we have decided to upload this as a separate Excel File: Supplementary Data 1. The legend (see cover Letter) provides explanation for each column/tab of this Excel file.*


---

* Your paper will be accompanied by a two-sentence editor's summary, of between 250-300 characters, when it is published on our homepage. Could you please approve the draft summary below or provide us with a suitably edited version.

"Notch signalling mediates intestinal enteroblast specification in Drosophila but the molecular mechanism as to how this is regulated is unclear. Here, the authors show that the transcription factor Klumpfuss regulates enteroblast-specific expression/repression of enteroendocrine cell fate downstream of Notch."

*We would suggest the following, slightly altered, summary:*

*"Notch signalling mediates intestinal enteroblast specification in Drosophila but the molecular mechanism as to how this is regulated is unclear. Here, the authors show that the transcription factor Klumpfuss ensures enteroblast commitment through repression of enteroendocrine cell fate downstream of Notch."*

* As part of our efforts to communicate our content to a wider audience, we endeavour to highlight papers published in Nature Communications on the journal’s Twitter account ([@NatureComms](https://twitter.com/NatureComms)). If you would like us to mention authors, institutions or lab groups in these tweets, please provide the relevant twitter handles in your cover letter upon resubmission.

*I would love to have myself included in a tweet (@JeromeKorzelius) and my current institute (@MPIAGE) as well*.

If you opted into the journal hosting details of a preprint version of your manuscript via a link on our dedicated website (<https://nature-research-under-consideration.nature.com>), it will remain on this site while you are revising your manuscript, as we consider the file to remain active. Should you wish to remove these details, please email [naturecommunications@nature.com](mailto:naturecommunications@nature.com) indicating your manuscript number and the link on our website that was previously sent to you. Please see our pre-publicity policy at <http://www.nature.com/authors/policies/confidentiality.html> For more information, please refer to our FAQ page at <https://nature-research-under-consideration.nature.com/posts/19641-frequently-asked-questions>

In recognition of the time and expertise our reviewers provide to Nature Communications’s editorial process, as of November, 2018, we formally acknowledge their contribution to the external peer review of articles published in the journal. All peer-reviewed content will carry an anonymous statement of peer reviewer acknowledgement, and for those reviewers who give their consent, we will publish their names alongside the published article. For more information, please refer to our FAQ page:
<https://www.nature.com/documents/ncomms-reviewer-information.pdf>

OPEN ACCESS:

Nature Communications is a fully open access journal. Articles are made freely accessible on publication under a [CC BY license](http://creativecommons.org/licenses/by/4.0) (Creative Commons Attribution 4.0 International License). This license allows maximum dissemination and re-use of open access materials and is preferred by many research funding bodies. 

For further information about article processing charges, open access funding, and advice and support from Nature Research, please visit <http://www.nature.com/ncomms/about/open-access>


SUBMISSION INFORMATION:

In order to accept your paper, we require the following:

* A cover letter describing your response to our editorial requests.

* A separate document detailing your point-by-point response to any issues raised by our referees (please include the referees' comments in this document).

* The final version of your text as a Word or TeX/LaTeX file, with any tables prepared using the Table menu in Word or the table environment in TeX/LaTeX and using the 'track changes' feature in Word.

* The complete author list provided in the article file, which must match that given on our manuscript tracking system. The author list in the main article file will be used during typesetting of your article.

* Production-quality versions of all figures, supplied as separate files containing all panels. To ensure the swift processing of your paper please provide the highest quality, vector format, versions of your images (.ai, .eps, .psd) where available. Please see our [brief guide to manuscript submission](https://www.nature.com/documents/ncomms-submission-guide.pdf) for further details on the figure formats we can accept. Text and labelling should be in a separate layer to enable editing during the production process. If vector files are not available then please supply the figures in whichever format they were compiled in and not saved as flat .jpeg or .TIFF files. Any chemical structures or schemes contained within figures should additionally be supplied as separate ChemDraw (.cdx) files. If your artwork contains any photographic images, please ensure these are at least 300 dpi.

To ensure that your figures are accessible to colour-blind readers, we encourage you to use alternative colour schemes. For example, rainbow colour scales may be replaced by single-colour intensity scales or greyscale, and red/green image overlays may be replaced with magenta/green. For reference an example of R-script colour blindness palettes can be found here <https://cran.r-project.org/web/packages/viridis/vignettes/intro-to-viridis.html.> Another example for Python can be found here: <http://matplotlib.org/cmocean/>

* The final version of any Supplementary Information (figures, tables, notes etc) in one PDF file. Please add a cover page to the Supplementary Information PDF, including the title of the manuscript and the first author's surname in the format ‘Smith et al.’ Please submit movies, audio files and data sets as separate files. See <http://www.nature.com/ncomms/submit/how-to-submit>#Supplementary-information for acceptable file formats/sizes.
** Please note that Supplementary Information must be finalised prior to acceptance of the paper. **  

* If you wish, an interesting image (but not an illustration or schematic) for consideration as a ‘Featured Image’ on the Nature Communications homepage. Examples can be seen on our Facebook page: <http://go.nature.com/PGPizM> The file should be 1400x400 pixels in RGB format and should be uploaded as 'Related Manuscript File'. In addition to our home page, we may also use this image (with credit) in other journal-specific promotional material.

* A completed author checklist, uploaded as a Related Manuscript file type, available at:
<https://www.nature.com/documents/ncomms-manuscript-checklist.pdf>

* Completed and signed copies of our Multimedia License to Publish (LTP) for any Featured Image suggestions (please use one form for each image and give a scientific description of the image in the 'title' field; do not use "Featured Image" as a title): 
[Multimedia Licence to Publish form](http://www.nature.com/documents/snl-multimedia-ltp.docx)

At acceptance, the corresponding author will be required to complete an Open Access Licence to Publish on behalf of all authors, declare that all required third party permissions have been obtained and provide billing information in order to pay the article-processing charge (APC) via credit card or invoice. 

Please note that your paper cannot be sent for typesetting to our production team until we have received these pieces of information; **therefore, please ensure that you have this information ready when submitting the final version of your manuscript.** Springer Nature encourages all authors and reviewers to adopt an Open Researcher and Contributor Identifier (ORCID). ORCID is a community-based initiative that provides an open, non-proprietary and transparent registry of unique identifiers to help disambiguate research contributions. All authors who link their ORCID to their account in our submission system will have their ORCID published on their articles. Please note that this is only possible if ORCIDs are linked prior to acceptance, that is, it is not possible to add ORCIDs at proof.

Please ensure that all co-authors are aware that they can link their ORCIDs, so that it will display on this paper. If they so wish, they must do so before the paper is formally accepted. It will not be possible to add ORCIDs post-acceptance, e.g. at proof. To link an ORCID please follow these instructions:

1. From the home page of the [MTS](https://mts-ncomms.nature.com/cgi-bin/main.plex) click on ‘Modify my Springer Nature account’ under ‘General tasks’.
2. In the ‘Personal profile’ tab, click on ‘ORCID Create/link an Open Researcher Contributor ID (ORCID)’. This will re-direct you to the ORCID website. 
3a. If you already have an ORCID account, enter your ORCID email and password and click on ‘Authorize’ to link your ORCID with your account on the MTS.
3b. If you don’t yet have an ORCID account, you can easily create one by providing the required information and then clicking on ‘Authorize’. This will link your newly created ORCID with your account on the MTS.

If you experience problems in linking your ORCID, please contact [Platform Support](mailto:platformsupport@macmillan.com)
